# *De novo* synthetic antimicrobial peptide design with a recurrent neural network

**DOI:** 10.1002/pro.5088

**Published:** 2024-07-11

**Authors:** Chenkai Li, Darcy Sutherland, Amelia Richter, Lauren Coombe, Anat Yanai, René L. Warren, Monica Kotkoff, Fraser Hof, Linda M. N. Hoang, Caren C. Helbing, Inanc Birol

**Affiliations:** ^1^ Canada's Michael Smith Genome Sciences Centre BC Cancer Agency Vancouver British Columbia Canada; ^2^ Bioinformatics Graduate Program University of British Columbia Vancouver British Columbia Canada; ^3^ Public Health Laboratory British Columbia Centre for Disease Control Vancouver British Columbia Canada; ^4^ Department of Pathology and Laboratory Medicine University of British Columbia Vancouver British Columbia Canada; ^5^ Department of Chemistry and the Centre for Advanced Materials and Related Technology University of Victoria Victoria British Columbia Canada; ^6^ Department of Biochemistry and Microbiology University of Victoria Victoria British Columbia Canada; ^7^ Department of Medical Genetics University of British Columbia Vancouver British Columbia Canada

**Keywords:** antibiotic resistance, antimicrobial peptide, *de novo* peptide design, recurrent neural network

## Abstract

Antibiotic resistance is recognized as an imminent and growing global health threat. New antimicrobial drugs are urgently needed due to the decreasing effectiveness of conventional small‐molecule antibiotics. Antimicrobial peptides (AMPs), a class of host defense peptides, are emerging as promising candidates to address this need. The potential sequence space of amino acids is combinatorially vast, making it possible to extend the current arsenal of antimicrobial agents with a practically infinite number of new peptide‐based candidates. However, mining naturally occurring AMPs, whether directly by wet lab screening methods or aided by bioinformatics prediction tools, has its theoretical limit regarding the number of samples or genomic/transcriptomic resources researchers have access to. Further, manually designing novel synthetic AMPs requires prior field knowledge, restricting its throughput. *In silico* sequence generation methods are gaining interest as a high‐throughput solution to the problem. Here, we introduce AMPd‐Up, a recurrent neural network based tool for *de novo* AMP design, and demonstrate its utility over existing methods. Validation of candidates designed by AMPd‐Up through antimicrobial susceptibility testing revealed that 40 of the 58 generated sequences possessed antimicrobial activity against *Escherichia coli* and/or *Staphylococcus aureus*. These results illustrate that AMPd‐Up can be used to design novel synthetic AMPs with potent activities.

## 1 INTRODUCTION

The worldwide overuse of antibiotics has created an alarming number of bacteria that possess antibiotic resistance, resulting in conventional antibiotics being less effective (Reardon, 2014). It is estimated that 1.27 million people died due to antibiotic resistance in 2019 (Antimicrobial Resistance Collaborators, 2022), and the speed of bacterial evolution, resulting in antibiotic resistance, is expected to greatly increase this death toll in the next few decades (Laxminarayan et al., 2013; O'Neill, 2014). Moreover, the sluggish pace of discovery and development of new therapeutics is exacerbating this public health crisis (Koo and Seo, 2019). As a result, novel effective substitutes for conventional antibiotics are urgently needed as weapons to fight against multidrug‐resistant bacteria, also referred to as “superbugs”.

Antimicrobial peptides (AMPs), a diverse class of short and often cationic peptides, are considered a viable alternative to conventional antibiotics (van der Does et al., 2019). Naturally occurring AMPs are observed among all forms of life (Zhang and Gallo, 2016). In higher eukaryotic organisms, AMPs have co‐evolved with environmental microbes as part of the host innate immune system (Zhang and Gallo, 2016). Microbes can also produce AMPs for inter‐competition purposes against the growth of other microbes (Zhang and Gallo, 2016). Most of the known AMPs reported in public databases are antibacterial, with some AMPs active or additionally active against other types of microbes (e.g., fungi, viruses) (Wang et al., 2016). Unlike most conventional antibiotics, which have specific functional or structural targets, most AMPs act directly on bacterial membranes or cell walls leading to non‐enzymatic disruption, with some eukaryotic AMPs performing additional modulation of the host immune system (Nguyen et al., 2011; Zhang and Gallo, 2016). As a result, it may be more difficult for bacteria to develop resistance to AMPs compared with conventional antibiotics (Boman, 2003). However, resistance to AMPs can still be observed if bacteria are exposed to AMPs for sufficient periods of time (Boman, 2003), indicating that antibiotic resistance is an enduring phenomenon. Thus, high‐throughput methods for the rapid discovery and design of novel AMPs would be instrumental in our fight against superbugs (Lin et al., 2022).

Recently, a number of *in silico* AMP prediction tools have been developed to reduce the labor and costs associated with large‐scale wet lab screening for AMP discovery (Jukič and Bren, 2022; Li et al., 2022; Meher et al., 2017; Veltri et al., 2018; Xiao et al., 2013). State‐of‐the‐art AMP prediction tools include AMPlify (Li et al., 2022), AMP Scanner Vr.2 (Veltri et al., 2018), iAMPpred (Meher et al., 2017), and iAMP‐2L (Xiao et al., 2013). Each of these tools utilizes machine learning methods, with AMPlify outperforming the latter three tools by adapting a deep learning model with attention mechanisms (Li et al., 2022; Vaswani et al., 2017; Yang et al., 2016). These *in silico* tools have successfully been applied in identifying novel, naturally occurring AMPs from genomic or transcriptomic resources (Li et al., 2022; Lin et al., 2022; Richter et al., 2022). Nevertheless, the discovery of these AMPs is limited by the availability of organism sources, such as tissue samples for direct wet lab screening or sequencing data for *in silico* mining. Even though *in silico* mining methods are high‐throughput, they require massive amounts of upstream work for careful data preparation (Li et al., 2022; Lin et al., 2022; Richter et al., 2022), which further limits the pace of development and the number of novel AMPs that can be discovered.

The potential sequence space of amino acids is combinatorially large, allowing for the design of peptide sequences that may not exist in nature but still possess desirable antimicrobial properties. Traditional approaches for AMP design include (1) modification of known AMP sequences to generate their congeners, fragments, or hybrids; (2) minimalist approaches by which AMPs are designed *de novo* purely based on structural requirements (e.g., amphipathic alpha‐helical structures) but with limited types (e.g., physicochemical properties) of residues used; (3) sequence‐template‐guided approaches that create sequence templates by comparing structurally homologous fragments from known AMPs for conserved patterns in terms of residue types; and (4) utilizing combinatorial peptide libraries (Huan et al., 2020; Tossi, 2011). However, these methods require prior expertise in AMPs' research for more accurate designs, which restricts the throughput.

Recently, a series of machine learning models based on neural networks have been proposed for the automatic *de novo* design of AMP sequences (Das et al., 2021; Dean et al., 2021; Gupta and Zou, 2019; Nagarajan et al., 2018; Szymczak et al., 2022; Tucs et al., 2020; Van Oort et al., 2021). They make it possible for users to sample novel AMP sequences directly from the models without any artificial design. Common sequence generation models include recurrent neural network (RNN) language models (Mikolov et al., 2010), variational autoencoders (VAEs) (Kingma and Welling, 2014), and generative adversarial networks (GANs) (Goodfellow et al., 2014). Nagarajan et al. developed a long short‐term memory (LSTM) RNN language model (Hochreiter and Schmidhuber, 1997; Mikolov et al., 2010), and embedded it into a framework with multiple filtering steps for the generation of novel AMPs with strong antibacterial activity (Nagarajan et al., 2018). Dean et al. proposed a VAE‐based AMP sequence generation framework, named PepVAE, for generation of highly active AMPs (Dean et al., 2021). Das et al. further adapted VAE and introduced CLaSS for controlled AMP sequence generation with attributes of interest (Das et al., 2021). HydrAMP, another VAE‐based model, incorporates two pre‐trained classifiers monitoring the quality of the generated peptides during training (Szymczak et al., 2022), improving upon a conditional VAE (cVAE) (Sohn et al., 2015). Gupta et al. proposed Feedback GAN for generating DNA sequences that encode proteins with optimized properties, and applied it to AMP sequence generation as an example (Gupta and Zou, 2019). Tucs et al. adapted an activity‐aware LeakGAN (Guo et al., 2018) to generate highly active AMPs (Tucs et al., 2020), while Van Oort et al. introduced AMPGAN v2 based on a bidirectional conditional GAN (BiCGAN) (Donahue et al., 2017; Dumoulin et al., 2017) to generate AMP sequences of different types and properties (Van Oort et al., 2021). The flurry of activities represented by these methods illustrate a strong interest in the field for *de novo* AMP design and explore expertise‐free approaches. Nonetheless, there is still room for improvement in generating AMP designs with desirable properties and high potency.

**FIGURE 1:**
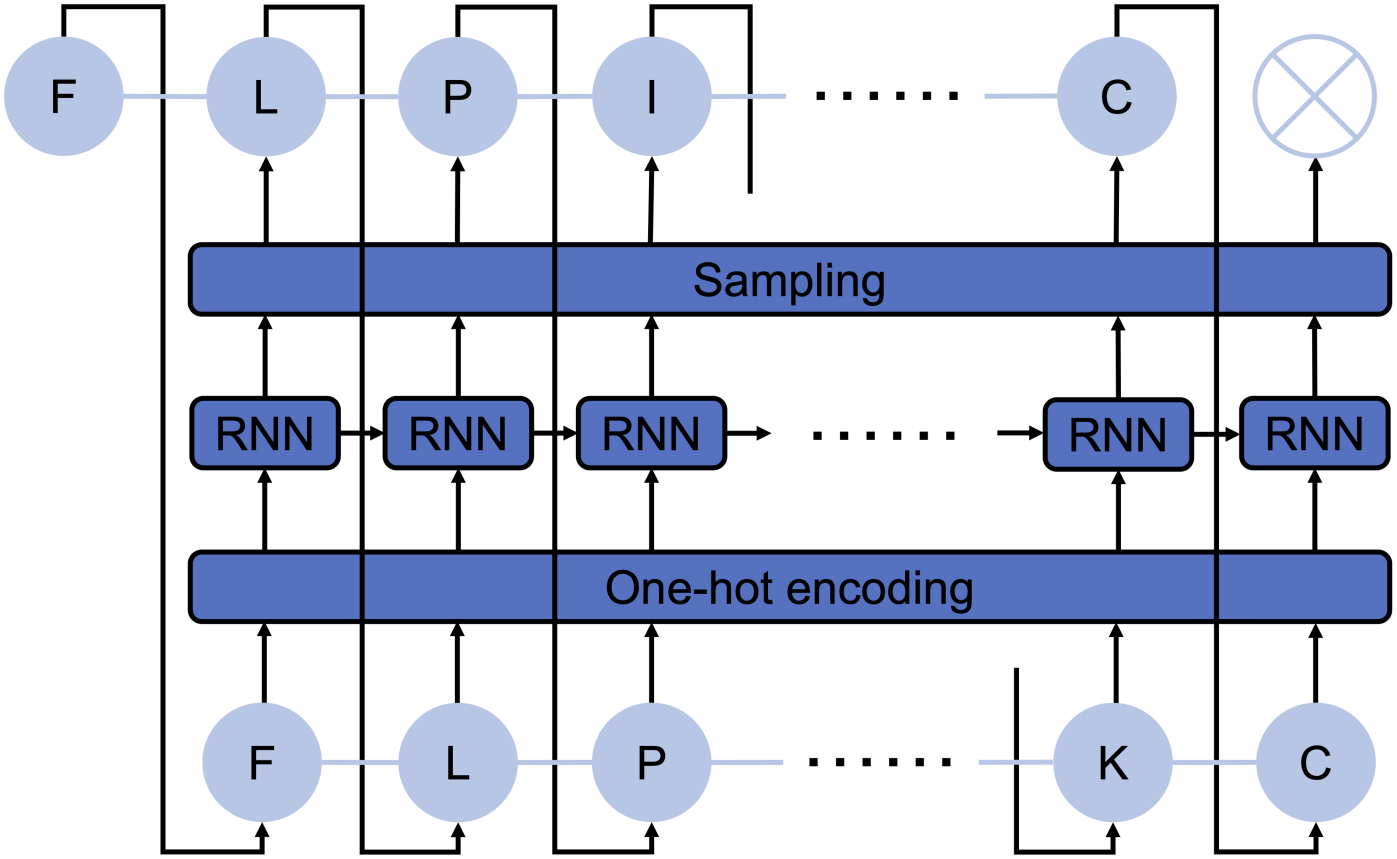
Architecture of the recurrent neural network (RNN) language model. Given a starting amino acid, the RNN language model predicts the next amino acids residue by residue until reaching the end‐of‐sequence (EOS) signal (represented as a cross marker). Amino acids, including the EOS signal, are one‐hot encoded. The output of RNN at each time step is a probability vector of amino acid and EOS occurrence at the next position, to which sampling strategies can be applied.

In the presented work, we introduce AMPd‐Up, a novel AMP sequence generation tool that implements a standard RNN language model (Mikolov et al., 2010) (Figure 1). The tool focuses on generating short AMP sequences ≤50 amino acids (aa) in length, with potential antibacterial activity. AMPd‐Up samples candidate AMP sequences from multiple model instances trained with different random initializations. For *de novo* AMP sequence generation, our RNN language model learns the “grammar”—the arrangement of the amino acids—of the training AMP sequences and estimates the probabilities of amino acid occurrence at each position recurrently starting from the N‐terminus. Thus, the model generates a putative AMP sequence, residue by residue, based on the probability distribution estimated at each residue position (or each time step of the process), until reaching the end‐of‐sequence (EOS) signal. We expect different model instances to capture the complicated underlying features of AMP sequences from slightly different aspects, thus exploring various localities in the state space represented by a rich repertoire of natural AMPs. With this approach, we generated 40 novel AMPs that have not been reported in public databases but were proven to be active against laboratory strains of *Escherichia coli* and/or *Staphylococcus aureus*. Our results illustrate the power of AMPd‐Up in contributing to our expanding arsenal of synthetic antimicrobial agents.

## 2 RESULTS

### 2.1 Performance comparison with state‐of‐the‐art methods

We measured the performance of AMPd‐Up by assessing the generated sequences using three state‐of‐the‐art AMP prediction tools: AMPlify (Li et al., 2022), AMP Scanner Vr.2 (Veltri et al., 2018), and iAMPpred (Meher et al., 2017). The estimated sequence generation accuracy values, expressed as the percentages of sequences predicted as AMPs by each AMP prediction tool, are reported in Table 1. The results of three other AMP sequence generation methods: the LSTM language model (Nagarajan et al., 2018), AMPGAN v2 (Van Oort et al., 2021), and HydrAMP (Szymczak et al., 2022), are listed in Table 1 for comparison. Although none of the *in silico* prediction tools are perfect in identifying AMPs, their reported performance (Li et al., 2022; Meher et al., 2017; Veltri et al., 2018) would be suitable for evaluating the AMP sequence generation methods. Details of how we calculated the estimated accuracy values can be found in Section 4.

**TABLE 1:** Performance comparison of different AMP sequence generation methods.

AMP sequence generation method	Estimated accuracy evaluated by AMP prediction tools (%)		
	By AMPlify	By AMP Scanner Vr.2	By iAMPpred
AMPd‐Up	**95.50 ± 0.35**	**100.00 ± 0.00**	**99.30 ± 0.37**
LSTM^a^	84.85 ± 0.75	84.20 ± 1.04	82.80 ± 0.97
AMPGAN v2^b^	90.90 ± 2.10	87.55 ± 1.29	94.85 ± 1.29
HydrAMP^c^	87.50 ± 1.15	94.60 ± 0.46	97.70 ± 0.64

*Note*: Different methods were evaluated using three *in silico* AMP prediction tools: AMPlify (Li et al., 2022), AMP Scanner Vr.2 (Veltri et al., 2018), and iAMPpred (Meher et al., 2017), based on sequences generated by each of the methods. The estimated AMP sequence generation accuracy measured by a selected prediction tool was defined as the percentage of peptide sequences predicted as AMPs among a generated sequence set. For each sequence generation method, five sets of sequences were generated, with 400 in each set. For each AMP sequence generation method, an average estimated accuracy value of the five generated sets was reported when measured by a specific AMP prediction tool, along with the corresponding standard deviation value. One‐sided Welch's *t*‐tests indicate that the superior performance of AMPd‐Up over its comparators is statistically significant (*p* < 0.05).

Abbreviations: AMP, antimicrobial peptide; LSTM, long short‐term memory.

^a^ Sequences sampled from the generated sequence set provided by the authors (Nagarajan et al., 2018).

^b^ Antibacterial peptides were selected for a fairer comparison with other methods (Van Oort et al., 2021).

^c^ Sequences generated using online server on November 7, 2022 (Szymczak et al., 2022).

As measured by AMPlify, AMPd‐Up obtains the highest estimated accuracy with 95.50% of the generated sequences predicted as AMPs on average, which outperforms the best comparator AMPGAN v2 by 4.60%, followed by HydrAMP (by 8.00%) and then the LSTM language model (by 10.65%). When evaluated using AMP Scanner Vr.2 and iAMPpred, AMPd‐Up generates AMP sequences with estimated accuracies of 100.00% and 99.30%, surpassing the best comparator HydrAMP by 5.40% and 1.60%, respectively. Although the rankings of the AMP sequence generation methods evaluated by the three AMP prediction tools are slightly different from each other, AMPd‐Up always performs the best compared with its comparators.

### 2.2 *De novo* generated sequences

Besides using the outputs of *in silico* AMP prediction tools as a proxy for performance, we also analyzed the generated sequences based on their amino acid compositions, length and net charge distributions, as well as their sequence similarity levels to the training set and all known AMP sequences. Details of how we analyzed the sequences generated by AMPd‐Up can be found in Section 4.

Figure S1 in Data S1 summarizes the amino acid compositions of the generated sequences. The sequences generated by AMPd‐Up were substantially rich in lysine (K) and leucine (L) residues, with average proportions of 29.87% and 24.51% per peptide sequence, respectively. In comparison, the sequences in our training set were rich in leucine (L), glycine (G), and lysine (K) residues, with average proportions of 11.50%, 10.94%, and 10.67%, respectively. Figure S1 in Data S1 additionally provides the amino acid composition information of the putative AMP sequences generated by three other methods. Two of the other methods (i.e., the LSTM language model and AMPGAN v2) highlighted lysine (K) and leucine (L) as predominant amino acid residues in their generated sequences, similar to the pattern observed in AMPd‐Up.

**FIGURE 2:**
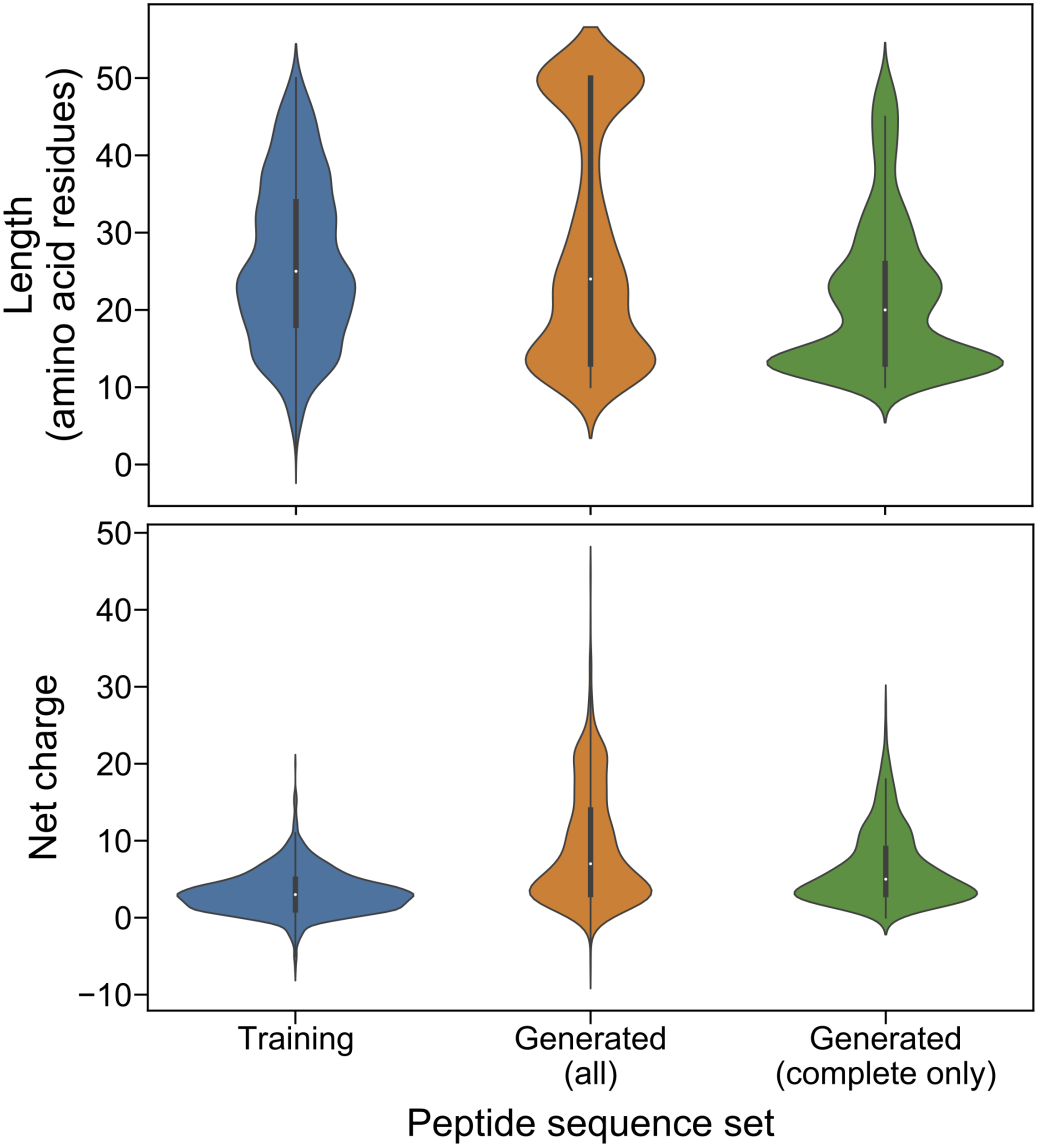
Length and net charge distributions of the sequences generated by AMPd‐Up. Length and net charge distributions were calculated based on 2000 sequences generated by AMPd‐Up, along with training sequences for comparison; 1484 of the 2000 generated sequences in “complete” status were chosen for an additional comparison. Mean (*μ*) and standard deviation (*σ*) of each distribution are as follows: training sequences (length: *μ* = 26.21 aa, *σ* = 10.34 aa; net charge: *μ* = 3.30, *σ* = 2.74), all generated sequences (length: *μ* = 28.90 aa, *σ* = 15.07 aa; net charge: *μ* = 9.08, *σ* = 7.33), and complete generated sequences (length: *μ* = 21.56 aa, *σ* = 9.87 aa; net charge: *μ* = 6.45, *σ* = 4.73).

Short lengths and net positive charges are common characteristics for most previously discovered AMPs (Zhang and Gallo, 2016), therefore many AMP studies investigate these key properties (Gagnon et al., 2017). Shorter peptides are also cheaper to synthesize (Lin et al., 2022), making translating shorter sequences for clinical application potentially more cost‐effective. Further, the net positive charges of cationic AMPs are responsible for the electrostatic interaction with the negatively charged bacterial membranes or cell walls (Zhang and Gallo, 2016), with studies illustrating that the antimicrobial activity of some AMPs can be improved by increasing their net charges (Zelezetsky and Tossi, 2006). The top section of Figure 2 compares the length distributions of the sequences generated by AMPd‐Up with those constituting the training set. We note that the model may fail to reach the EOS signals when generating some sequences (referred to as “incomplete sequences”; see Section 4 for details); we thus additionally compared the generated sequence set with those incomplete sequences removed. The average generated sequence length was 28.90 aa, but was reduced to 21.56 aa after incomplete sequences were removed. The incomplete sequences are 50 aa by default. The complete sequences were 4.65 aa shorter than the training sequences on average. The bottom section of Figure 2 shows a similar comparison for net charge distributions. The average generated sequence net charge was 9.08, but was reduced to 6.45 after incomplete sequence removal. However, the net charge of the complete sequences was still 3.15 greater than the training sequences on average.

**FIGURE 3:**
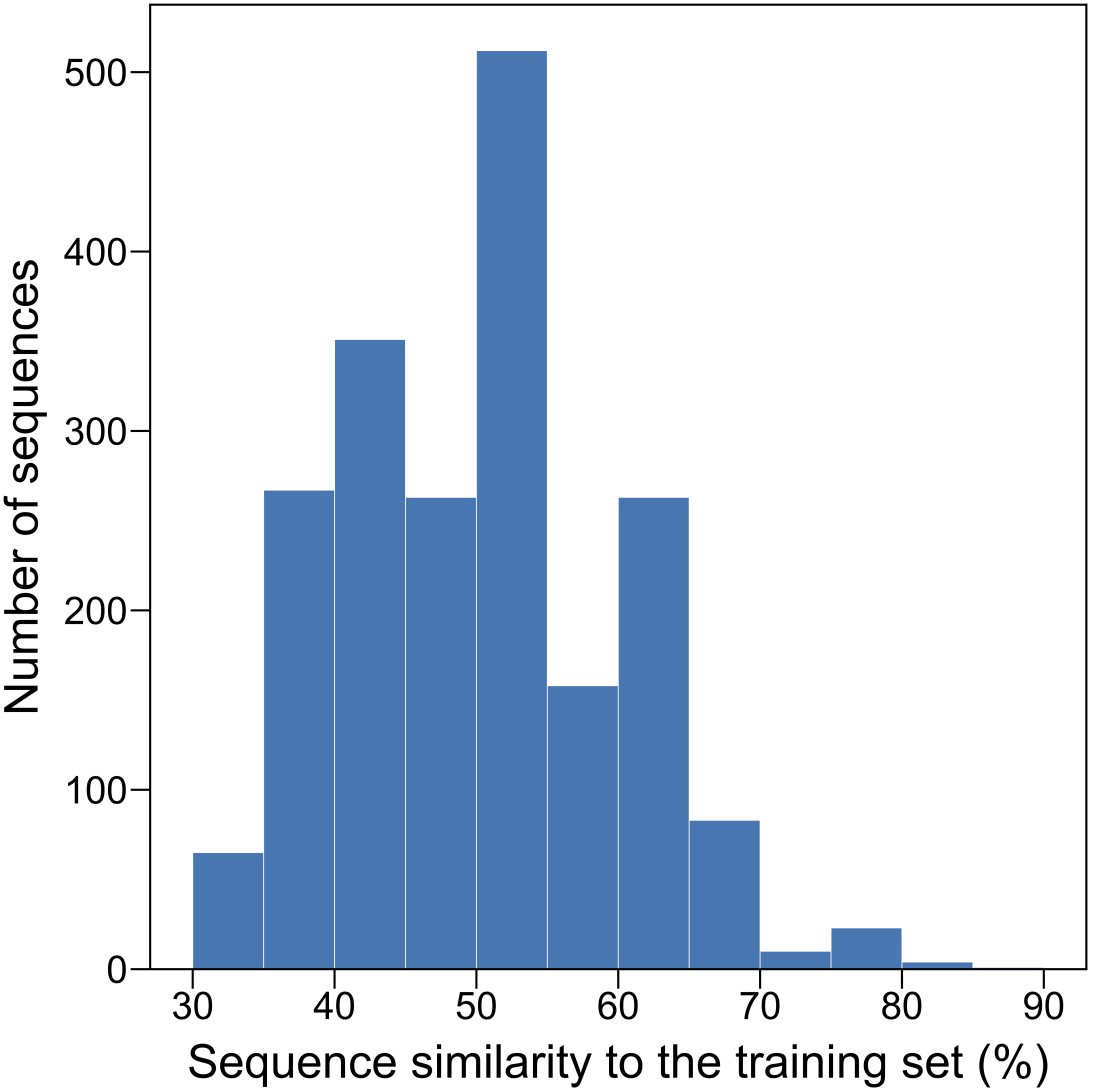
Sequence similarity distribution of the AMPd‐Up‐generated sequences to the training set. The sequence similarity distribution, with a mean of 49.97% and a standard deviation of 9.83%, was calculated based on the 2000 sequences generated by AMPd‐Up. The sequence similarity of each generated sequence to the training set was considered as the similarity of that sequence to its most similar sequence in the training set, based on which the distribution was plotted.

The sequence similarity of each AMPd‐Up‐generated sequence to the training set, composed of antibacterial peptides, was calculated for analysis (details in Section 4). Figure 3 shows the sequence similarity distribution of the AMPd‐Up‐generated sequences to the training set, with a peak between 50.00% and 55.00%. The generated sequences possess a similarity level of 49.97% compared with the training sequences on average, indicating that AMPd‐Up generates novel AMP sequences different from the training sequences. This implies that AMPd‐Up may be capturing high‐level features of AMPs, rather than only memorizing sequence‐level information during training. An additional test on the sequence similarity of each AMPd‐Up‐generated sequence to all available known AMPs from Antimicrobial Peptide Database (APD3, https://aps.unmc.edu) (Wang et al., 2016) and Database of Anuran Defense Peptides (DADP, http://split4.pmfst.hr/dadp) (Novković et al., 2012) was done (details in Section 4), with an average sequence similarity level of 51.03%, indicating the novelty of our generated sequences as compared with known AMPs (Figure S2 in Data S1). To supplement the sequence similarity analysis, we also visualized the pairwise sequence similarities between different sequence sets (Figure S3 in Data S1). A lower generated sequence similarity level between different model instances of AMPd‐Up (33.56%) than within the same model instance (39.14%) indicates that different model instances tend to capture features of AMPs from slightly different aspects. We expect the novelty of generated sequences by our tool to add diversity to the current AMP sequence databases.

### 2.3 *In vitro* validation results

We selected 58 peptide sequences, generated by 1000 AMPd‐Up model instances, for *in vitro* validation and bioactivity assessment. We organized our candidates into three lists: List A (DeNo1001 to DeNo1038) and List B (DeNo1039 to DeNo1042) were sampled through AMPd‐Up scores, and 16 more sequences that appeared with high frequencies of ≥40 in the generated set were selected to make List C (DeNo1043 to DeNo1058). AMPd‐Up score ranges from 0 to 1 and is a measure of the confidence level of the model when generating the sequence (see Section 4 for detailed definition). Table 2 summarizes the sequence specifications of our 58 selected putative AMPs. All sequences in Lists A and C were predicted as AMPs by AMPlify, while all sequences in List B were predicted as non‐AMPs.

**TABLE 2:** Putative AMP sequences generated by AMPd‐Up that have been prioritized for synthesis.

List	Peptide name	Sequence	# aa	Net charge^a^	Molecular weight (Da)	AMPd‐Up score^b^	AMPlify score^c^	Count^d^	Sequence similarity to known^e^ (%)
A	DeNo1001	DLLSGLGKAAKKVAKTVLKNLLKC	24	5	2512.12	0.2362	80.00	1	45.83
	DeNo1002	NLLDTLKNLAKKLAKKLLKKLLKKL	25	8	2889.71	0.2626	80.00	1	51.61
	DeNo1003	NLLSTLLDAAKKAAKGAAKSAAKKLAKKLAKKL	33	9	3364.14	0.2679	80.00	1	48.48
	DeNo1004	HLLSGLLSAAKKAAKKAAKKALKKLLKKLLKKL	33	12	3553.57	0.2891	80.00	1	45.45
	DeNo1005	GLFSLLKKLLKKLLKKLLKKLLKKLLKKL	29	12	3431.61	0.2942	80.00	1	58.62
	DeNo1006	NLLDTLKKKAKKVAKKVLKKLLKKLLKKL	29	12	3373.40	0.3150	80.00	1	48.48
	DeNo1007	FLPSIIKGAAKKLPKIFCKILKKC	24	7	2688.49	0.3313	80.00	1	75.00
	DeNo1008	GLLSLLKKLLKKLLKKLLKKL	21	8	2432.27	0.2949	80.00	11	66.67
	DeNo1009	DLLKTLGKAAKKAAKTALKAALKGLLKKLAKKL	33	10	3446.37	0.2467	69.24	1	45.45
	DeNo1010	VLGGLLKKLLKKLLKKL	17	6	1905.55	0.2520	69.24	1	58.82
	DeNo1011	HLLSLLKKAAKKLLKKLLKKLAKKL	25	10	2868.78	0.3049	69.24	1	48.00
	DeNo1012	CLLDTLKCVAKGVAGTLLDTLKCKITGKC	29	3	3009.73	0.2732	67.48	1	70.00
	DeNo1013	KIFGKILKKLLKKLLKKLLKKL	22	10	2635.55	0.2995	64.47	1	54.55
	DeNo1014	ALPSLLKKLAKKLAKKLLKKLLKKLLKKLLKKL	33	14	3794.08	0.3100	64.47	1	48.48
	DeNo1015	NLLDTLKNVAKNVAKNVLDTLKCKITCKC	29	4	3204.89	0.3346	64.47	1	65.52
	DeNo1016	FLPIIAGLAAKFLPKIFCKITKKC	24	5	2664.42	0.3579	64.47	1	87.50
	DeNo1017	FLPIIAGLAAKLLPKLFCKITKKC	24	5	2630.41	0.3519	60.79	1	87.50
	DeNo1018	WLPKIAGKIAGKLLKKLLKKIKKK	24	10	2744.60	0.2573	60.21	1	50.00
	DeNo1019	FLPKIAGKAAKKLPKIFCKITKKC	24	8	2675.45	0.3314	58.82	2	83.33
	DeNo1020	TLPDVAKNVAKNVAKTVLDTLKCKITGKC	29	4	3072.70	0.3259	58.45	1	75.86
	DeNo1021	KLFGKLLKKLKKILKKIAKKIKKKL	25	13	2977.99	0.2309	57.62	1	44.83
	DeNo1022	GLLSLLKKIGKKIGKLL	17	5	1822.38	0.2415	55.72	1	58.82
	DeNo1023	DLLKTLKKIAKKLLKTLLKKLLKKLLKKL	29	11	3415.52	0.2838	53.67	1	48.28
	DeNo1024	KLFGKILGKIAKKILGKILGALLSKLLSAL	30	7	3149.05	0.2150	53.22	1	46.67
	DeNo1025	DLLSCLKKKGKCVLKNL	17	4	1903.41	0.1885	49.48	1	47.06
	DeNo1026	RLPSLFKKLFKKIAKVVGKIAKKILKK	27	11	3124.05	0.2203	48.34	1	45.71
	DeNo1027	RLPSIIPGIAGKLGGLLGGLLKGL	24	3	2313.88	0.2044	43.84	1	54.17
	DeNo1028	CLPSLLPSLFKKL	13	2	1458.86	0.1713	34.88	3	53.85
	DeNo1029	SLPSILSGIAGKL	13	1	1255.51	0.1991	32.75	1	61.54
	DeNo1030	RLPRIFRGIRGKL	13	5	1581.96	0.1674	31.93	1	46.15
	DeNo1031	PLPPIIPGIAGKLLGGLLGLLKKL	24	3	2392.07	0.2256	31.81	1	54.17
	DeNo1032	YLPSVLPSVLKPL	13	1	1425.76	0.1779	31.78	1	53.85
	DeNo1033	PLPPIIPGLASGLLSGLC	18	0	1718.12	0.1938	24.72	1	61.11
	DeNo1034	KLPSIIKAAAKALPKLF	17	4	1809.30	0.2097	20.81	1	52.94
	DeNo1035	QLPRIAGKIAKKL	13	4	1435.81	0.1833	17.41	1	61.54
	DeNo1036	QLPSVLPAIAKAL	13	1	1320.63	0.1609	9.02	1	53.85
	DeNo1037	CLPSILC	7	0	747.97	0.1462	7.88	1	41.67
	DeNo1038	MLPSIAGAAAKGLPKLFCKITKKC	24	5	2490.16	0.2784	3.88	1	79.17
B	DeNo1039	MLPKIFGKIFKKILKKILKKILKKILKKLLKKL	33	14	3976.37	0.3091	1.32	1	42.42
	DeNo1040	MLPSILGALLKLL	13	1	1381.82	0.2000	0.83	3	61.54
	DeNo1041	MLPKIAGKIAKKL	13	4	1410.86	0.2210	0.80	62	61.54
	DeNo1042	MLPKIAGAIAKLL	13	2	1338.75	0.2080	0.77	8	61.54
C^f^	DeNo1043	WLPKIAGKIAGKL	13	3	1394.75	0.2202	19.83	95	61.54
	DeNo1044	CLPSILCKITKKC	13	3	1449.90	0.2147	35.64	90	53.85
	DeNo1045	FLPKIFKKIAKKL	13	5	1574.06	0.2659	25.78	77	61.54
	DeNo1046	VLGSLLKGLLKKL	13	3	1381.80	0.2240	80.00	65	61.54
	DeNo1047	ALPSIIKGLLKKL	13	3	1393.81	0.2104	53.55	63	53.85
	DeNo1048	LLPSLLKGLLKKL	13	3	1435.89	0.2282	56.01	58	61.54
	DeNo1049	ALLSLLKKLLKKL	13	4	1480.97	0.2426	69.24	56	61.54
	DeNo1050	FLPKIAGKIAGKL	13	3	1355.72	0.2400	10.81	55	69.23
	DeNo1051	ALPSLLKKLLKKL	13	4	1464.93	0.2259	54.39	50	53.85
	DeNo1052	YLPSVLKGLLKKL	13	3	1471.88	0.2032	46.71	48	53.85
	DeNo1053	LLPSLLKGLAKKL	13	3	1393.81	0.2267	52.80	47	61.54
	DeNo1054	QLPKIAGKIAKKL	13	4	1407.79	0.2159	12.97	47	61.54
	DeNo1055	FLPKIFKKIAKKI	13	5	1574.06	0.2514	39.72	46	55.56
	DeNo1056	GLLSLLKKLLKKL	13	4	1466.94	0.2490	80.00	43	69.23
	DeNo1057	ILGKLLKKLLKKL	13	5	1508.04	0.2325	51.38	40	61.54
	DeNo1058	FLPKIAGKIAKKL	13	4	1426.84	0.2474	19.60	40	69.23

*Note*: Lists A, B, and C include 38, 4, and 16 sequences, respectively. All sequences in Lists A and C were predicted as AMPs by AMPlify (Li et al., 2022), while those in List B were predicted as non‐AMPs. Sequences were sampled from all candidate peptide sequences generated by 1000 model instances, with incomplete sequences removed. Sequences in Lists A and B were sampled through AMPd‐Up scores, while List C comprises a set of sequences that appeared with high frequencies (≥40 in sequence counts) in all candidate peptides. The numbering of peptide names for Lists A and B was by AMPlify score, while List C was by sequence count, both in descending order.

Abbreviation: AMP, antimicrobial peptide.

^a^ Net charge at pH = 7.

^b^ AMPd‐Up scores range from 0 to 1; average AMPd‐Up score was reported for the same sequence generated by multiple model instances.

^c^ AMPlify scores range from 0 to 80; sequences with AMPlify scores >3.01 (i.e., AMPlify probability scores >0.5) are predicted as AMPs.

^d^ Frequency of the sequence appearing in the generated set.

^e^ Sequence similarity to the most similar known AMP sequence from Antimicrobial Peptide Database (APD3) (Wang et al., 2016) and Database of Anuran Defense Peptides (DADP) (Novković et al., 2012).

^f^ Sequences with shorter lengths (≤20 aa) but higher net charges (≥+3) were prioritized for List C.

**FIGURE 4:**
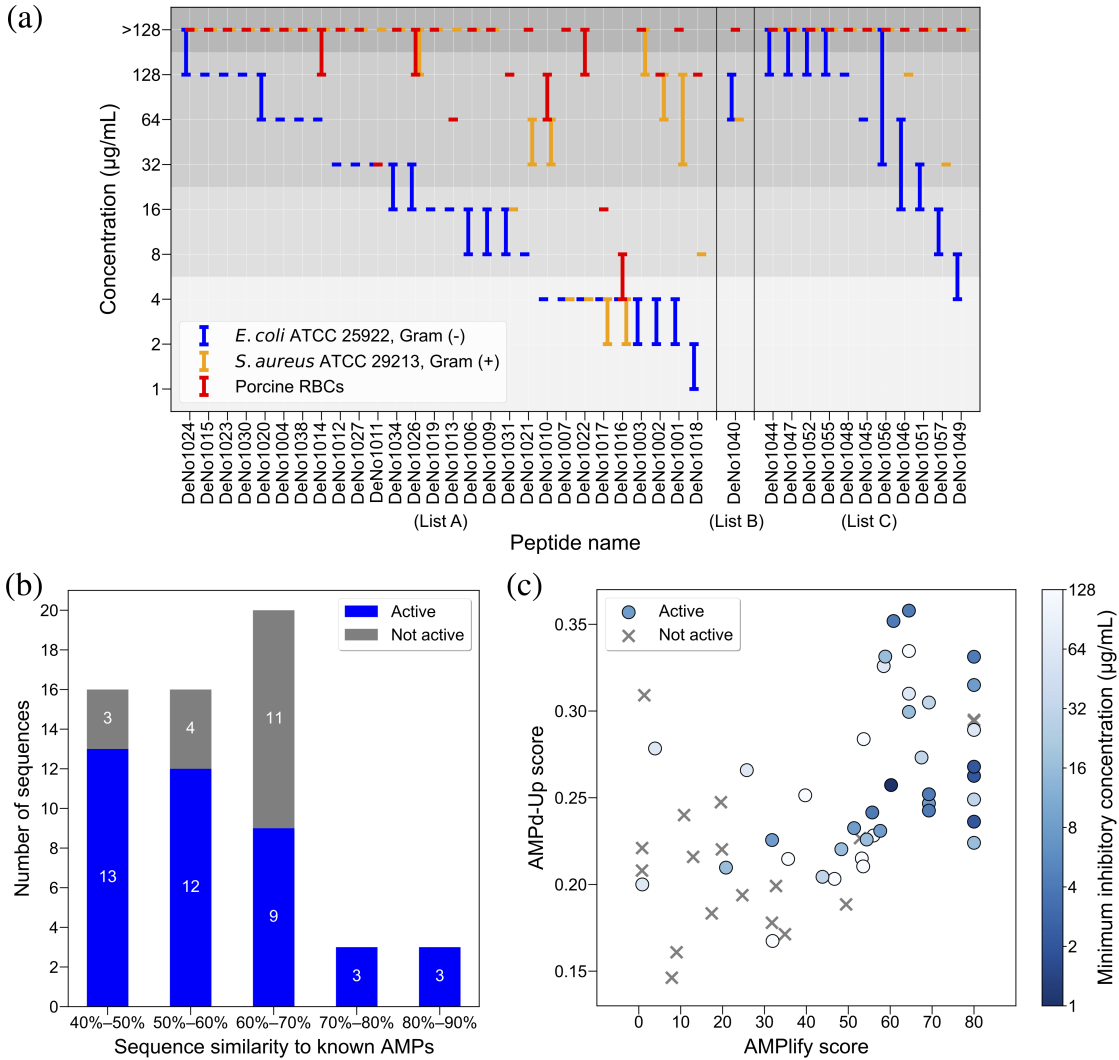
*In vitro* validation results of the 58 selected putative AMPs. (a) Antimicrobial and hemolytic activities of the 40 peptides that were active against at least one bacterial strain of *Escherichia coli* ATCC 25922 and *Staphylococcus aureus* ATCC 29213. Antimicrobial and hemolytic activities were measured by minimum inhibitory concentration (MIC) and concentration that lyses 50% (HC_50_) of the red blood cells (RBCs), respectively. HC_50_ was determined using porcine RBCs. Data are presented as the lowest effective peptide concentration range (μg/mL) observed in three independent experiments performed in duplicate, with one maximum data point and one minimum data point dropped for each measurement. The three sections from left to right correspond to peptides with observable antimicrobial activity from List A (*n* = 28), List B (*n* = 1), and List C (*n* = 11), respectively. Activity of the peptides was split into four levels: high (≤4 μg/mL), moderate (8–16 μg/mL), low (32–128 μg/mL), and without observable activity (>128 μg/mL), as separated by different background colors in the plot. (b) Stacked bar chart showing proportions of peptides that displayed antimicrobial activity with different sequence similarity levels to known AMPs from Antimicrobial Peptide Database (APD3) (Wang et al., 2016) and Database of Anuran Defense Peptides (DADP) (Novković et al., 2012). All similarity ranges are left‐open and right‐closed, and the sequence similarity of each candidate peptide to known AMPs was considered as the sequence similarity of that sequence to its most similar known AMP sequence. (c) Visualization of antimicrobial activity of the 58 tested peptides with respect to AMPlify (*x*‐axis) and AMPd‐Up (*y*‐axis) scores. AMPd‐Up scores of the same peptide sequences generated by multiple model instances were averaged. Peptides without any observable antimicrobial activity are presented as gray crosses, and the active peptides are presented as blue dots. Dots with darker colors indicate stronger antimicrobial activity against *Escherichia coli* ATCC 25922, determined by the lowest MIC value of each peptide against the strain. AMP, antimicrobial peptide; RBC, red blood cell.

Our 58 candidate peptides were tested against two bacterial isolates: the Gram‐negative *E. coli* ATCC 25922 and the Gram‐positive *S. aureus* ATCC 29213. Porcine red blood cells (RBCs) were used to assess the hemolytic activity of the peptides. Out of the 58 peptides selected for *in vitro* validation, 40 peptides displayed antimicrobial activity against at least one bacterial strain tested. All 15 peptides that were active against *S. aureus* ATCC 29213 also showed antimicrobial activity against *E. coli* ATCC 25922. Figure 4a visualizes the antimicrobial and hemolytic activities of the 40 peptides, in minimum inhibitory concentration (MIC) and concentration that lyses 50% of the RBCs (HC_50_), respectively. The entire *in vitro* validation results of the 58 peptides are shown in Table S1 in Data S1. For a better interpretation of the results, we split the activity of the tested peptides into four levels according to the MIC/HC_50_ ranges: high (≤4 μg/mL), moderate (8–16 μg/mL), low (32–128 μg/mL), and without observable activity (>128 μg/mL).

Among the 38 List A peptides tested, 28 peptides displayed antimicrobial activity, 12 of which were active against both strains tested (Figure 4a). Nine of the List A peptides were highly active against *E. coli* ATCC 25922, with DeNo1018 being the most active with an MIC of 1–2 μg/mL. These same nine peptides were also active against *S. aureus* ATCC 29213. Four of the nine peptides were highly active against *S. aureus* ATCC 29213 (MIC = 2–4 μg/mL for DeNo1016 and DeNo1017; MIC = 4 μg/mL for DeNo1007 and DeNo1022), with one (DeNo1018) moderately active (MIC = 8 μg/mL). Six peptides from List A were moderately active against *E. coli* ATCC 25922, and another two showed low to moderate activity against the strain. Three of these eight peptides displayed some antimicrobial activity against *S. aureus* ATCC 29213, one of which (DeNo1031) was moderately active (MIC = 16 μg/mL) with the other two (DeNo1021 and DeNo1026) showed low (MIC = 32–64 μg/mL) and minimal activity (MIC ≥ 128 μg/mL), respectively. Among all 28 List A peptides with proven antimicrobial activity, three were minimally hemolytic (HC_50_ ≥ 128 μg/mL) and 17 did not show any hemolytic activity (HC_50_ > 128 μg/mL) in our tests. DeNo1007 was the only AMP with high antimicrobial activity against both bacterial strains tested (MIC = 4 μg/mL) and without observable hemolytic activity (HC_50_ > 128 μg/mL).

Among the four peptides from List B tested, only DeNo1040 displayed some low‐level activity against the two bacterial strains tested (Figure 4a). Specifically, this peptide inhibited the growth of *E. coli* ATCC 25922 and *S. aureus* ATCC 29213 providing MICs of 64–128 μg/mL and 64 μg/mL, respectively. DeNo1040 also did not show any hemolytic activity in our tests (HC_50_ > 128 μg/mL). We note again that peptides in List B were predicted as non‐AMPs by AMPlify.

Among the 16 List C peptides tested, a total of 11 peptides showed antimicrobial activity against *E. coli* ATCC 25922, with two of them additionally active against *S. aureus* ATCC 29213 (Figure 4a). DeNo1049 displayed moderate to high activity against *E. coli* ATCC 25922 (MIC = 4–8 μg/mL), which was the strongest in List C. DeNo1057 was moderately antibacterial against *E. coli* ATCC 25922 (MIC = 8–16 μg/mL), followed by DeNo1051 (MIC = 16–32 μg/mL) and DeNo1046 (MIC = 16–64 μg/mL). DeNo1057 and DeNo1046 were the only two List C peptides with antibacterial activity against *S. aureus* ATCC 29213, though with low activity (MIC = 32 μg/mL and 128 μg/mL, respectively). None of the List C peptides displayed hemolytic activity (HC_50_ > 128 μg/mL).

Among the peptides that did not show any antimicrobial activity against the bacterial strains tested, most of them were also not hemolytic to the porcine RBCs except DeNo1008 (HC_50_ = 16–32 μg/mL) and DeNo1039 (HC_50_ = 32–64 μg/mL) as shown in Table S1 in Data S1.

In summary, List A has the largest proportion (73.68%) of putative AMPs observed with antimicrobial activity in our tests, followed by List C (68.75%) and List B (25.00%). Figure 4b presents the proportions of peptides that were active against at least one of the bacterial strains tested under different sequence similarity levels to the known AMPs from APD3 (Wang et al., 2016) and DADP (Novković et al., 2012). All six peptides between sequence similarities of 70.00% and 90.00% to known AMPs showed antimicrobial activity in our tests. The largest proportion of the tested peptides fall between sequence similarities of 60.00% and 70.00% to known AMPs, with nine out of 20 sequences displaying antimicrobial activity. Interestingly, lower similarity intervals of 50.00%–60.00% and 40.00%–50.00% possess relatively high proportions of antimicrobially active peptides with rates of 75.00% (12/16) and 81.25% (13/16), respectively. More than half (62.50%) of the peptides with antimicrobial activity from our experiments fall into these intervals, implying there is much to be explored in the sequence space for novel AMPs. Figure 4c visualizes the distribution of the 58 tested putative AMPs with regard to AMPlify scores and AMPd‐Up scores. AMPlify score, ranging from 0 to 80, is a prediction score reported by AMPlify, which is a log transformation of the AMPlify probability score $p_{\text{AMPlify}}$ as $-10\log_{10}\left(1-p_{\text{AMPlify}}\right)$. Considering the fact that multiple model instances may generate the same sequence but with different AMPd‐Up scores, the average was taken in the visualization for a more comprehensive analysis. As evident in Figure 4c, most of the peptides without any observable antimicrobial activity in our tests are located at the bottom left of the figure, suggesting that it is a viable strategy to prioritize generative sequences with both high AMPlify and AMPd‐Up scores for *in vitro* validation assays.

## 3 DISCUSSION

In the presented work, we introduce AMPd‐Up, a tool for *de novo* AMP sequence generation. AMPd‐Up adopts an RNN language model, sampling from multiple model instances trained with different random initializations. AMPd‐Up is available online as an open‐source tool at https://github.com/bcgsc/AMPd-Up. Although the architecture of our model is relatively simple compared with existing methods, we show that simple models like AMPd‐Up can work well if properly trained. The simplicity of our model architecture also brings with it lower computational costs. Moreover, the sequences generated by AMPd‐Up are of high novelty compared with existing AMP sequences in public databases, demonstrating the ability of our model to learn high‐level AMP features.

While AMPd‐Up shows great promise and favorable performance, the size of its training set is still relatively small (2253 sequences) compared with that of many traditional machine learning tasks for broader sequence data analysis, such as sentiment analysis or machine translation, which typically use hundreds of thousands to millions of data points for training available through public databases (Khurana et al., 2023). Furthermore, AMPd‐Up does not take the strength of antimicrobial activities (i.e., MIC values) into consideration during training. The MIC values of an AMP against the same bacterial strain may vary due to the differences in protocols utilized across different laboratories (Schuurmans et al., 2009), thereby diminishing the comparability of those values within existing public AMP databases. We expect these limitations to be gradually resolved as the ongoing discovery and validation of AMPs is bringing more high‐quality and well‐organized data, leading to further improvement in *de novo* AMP sequence generation tools like AMPd‐Up.

Although the AMPd‐Up‐generated putative AMPs have a considerable level of sequence diversity (Figure S3 in Data S1), we still noticed some patterns at the sequence level. Analyzing a set of 20,000 generated sequences, we observed that “LLKK” and “LKKL” were the two most frequently occurring 4‐mer motifs, appearing in 44.09% and 40.73% of the generated sequences, respectively. Previous studies have shown that synthetic amphipathic alpha‐helical peptides made up of repeat units [LLKK]_*n*_ or [LKKL]_*n*_ have antimicrobial properties (Khara et al., 2017; Wiradharma et al., 2011), which can explain these findings to some extent. In fact, it is suggested that repeats of 4‐mer units such as these are responsible for the formation of cationic amphipathic alpha‐helical structures, a key initiating step to the bioactivity and membrane‐disrupting properties of many AMPs (Khara et al., 2017; Wiradharma et al., 2011).

Among the 58 novel putative AMP sequences generated by AMPd‐Up, 40 showed antimicrobial activity, 15 of which were broadly antibacterial against both Gram‐positive and Gram‐negative isolates. Promisingly, one of the most active peptides, DeNo1007, not only possessed high antimicrobial activity against the two bacterial strains tested, but was also without observable hemolytic activity. We expect the AMP candidates generated by AMPd‐Up to increase the diversity of known peptide‐derived antibiotics, currently populated by mostly naturally occurring sequences, and to augment the candidate set of potential alternatives to conventional antibiotics. Although some of our putative AMPs did not show any antimicrobial activity against the two bacterial strains tested *in vitro*, they may still be active against other bacterial species and/or possess unexplored modes of action. Also, the structures of some AMPs may vary based on their microenvironment (Cândido et al., 2019). Further experimentation could be done to test candidate sequences on a wider panel of bacterial species, to investigate the variances in their antimicrobial mechanisms against bacteria with different membrane and cell wall structures (e.g., Gram‐positive vs. Gram‐negative bacteria), or to interrogate *in vivo* biological interactions.

Results from work like ours also have broader potential impact. Resistance to last‐line peptide‐based therapeutics, such as colistin and other polymyxins, is increasingly being reported (Aghapour et al., 2019). Concerningly, this is sometimes presented with cross‐resistance to multiple AMPs (Fleitas and Franco, 2016), highlighting the need for multiple and diverse classes of peptide‐based antimicrobials. *De novo* AMP sequence generation provides a rational solution to this problem, as one would theoretically expect that pathogens would be naïve to many of the diverse *de novo* generated AMPs. Even though there may be natural AMPs similar to some of the *de novo* generated ones, the vast sequence space of amino acids (e.g., 10^20^ or one hundred quintillion for a 10‐residue peptide sequence) virtually ensures that there would be a practically infinite number of them out there that are “new” to most common pathogens. Thus, we expect high‐throughput *in silico* AMP sequence design tools like AMPd‐Up to play a vital role in the fight against antibiotic resistance and the imminent rise of antibiotic‐resistant bacteria.

## 4 MATERIALS AND METHODS

### 4.1 Training set

To get our RNN language model well trained, a curated set of known AMP sequences are required to comprise the training set. Our work primarily focused on AMPs with direct antibacterial activity, a major function of most known AMPs. We also limited the generated AMP sequences to include only standard amino acids with a maximum length of 50 aa, reflecting the fact that most documented AMPs are relatively short (Zhang and Gallo, 2016).

All antibacterial peptide sequences were downloaded from APD3 (Wang et al., 2016) on March 20, 2019, a manually curated and annotated database for AMPs. This set of sequences contained 2571 AMP records with antibacterial activity, 2276 of which were ≤50 aa long. After removing duplicates and sequences with non‐standard amino acids, we ended up with a non‐redundant set of 2253 antibacterial sequences ≤50 aa in length, forming the training set for our RNN language model.

### 4.2 Model architecture and implementation

The implementation of the RNN language model was adapted from the PyTorch online tutorial by Sean Robertson (Robertson, 2017), with PyTorch library 1.7.1 (Paszke et al., 2019) in Python 3.6.7. During the training process, cross‐entropy was used as the loss function, and stochastic gradient descent (Robbins and Monro, 1951) was applied to optimize the model weights. We also adopted dropout technique (Srivastava et al., 2014) to prevent overfitting. The hyperparameters, which cannot be learned directly from training, were tuned through stratified five‐fold cross‐validation on the training set. The set of hyperparameters for model architecture and training settings with the lowest average cross‐validation loss was determined to be the optimal one to train the final model.

Figure 1 shows the architecture of the RNN language model, represented as a chain of repeating RNN cells. Given the first N‐terminal amino acid, the RNN language model generates a peptide sequence residue by residue until reaching the EOS signal. In this specific task of AMP sequence generation, we set the maximum length to be 50 and only the 20 standard amino acids are considered. Amino acids, together with the EOS signal, are encoded as 21 distinct one‐hot vectors, with $x_{t}\in {\mathbb{R}}^{21}$ representing the *t*‐th residue of a generated sequence. In this task, a time step *t* is defined as the process of an RNN cell predicting the $\left(t+1\right)$‐th residue $x_{t+1}$ of a sequence. At each time step *t* of the generation process, the RNN cell takes the hidden state $h_{t-1}$ from the previous time step and the predicted amino acid for the t‐th residue $x_{t}$ as input, and outputs a set of probabilities $p_{t}$ of amino acid and EOS occurrence at the next position, from which $x_{t+1}$ can be sampled. The hidden state $h_{t}\in {\mathbb{R}}^{d_{h}}$ and probability vector $p_{t}\in {\mathbb{R}}^{21}$ at each time step are calculated as:

$$h_{t}=W_{h}\left[\begin{array}{c} x_{t}\\ h_{t-1} \end{array}\right]+b_{h},$$

$$p_{t}=\text{softmax}\left(W_{p}\left[\begin{array}{c} h_{t}\\ W_{o}\left[\begin{array}{c} x_{t}\\ h_{t-1} \end{array}\right]+b_{o} \end{array}\right]+b_{p}\right),$$

where $W_{h}\in {\mathbb{R}}^{d_{h}\times \left(d_{h}+21\right)}$, $W_{o}\in {\mathbb{R}}^{21\times \left(d_{h}+21\right)}$, and $W_{p}\in {\mathbb{R}}^{21\times \left(d_{h}+21\right)}$ are weight matrices, and $b_{h}\in {\mathbb{R}}^{d_{h}}$, $b_{o}\in {\mathbb{R}}^{21}$, and $b_{p}\in {\mathbb{R}}^{21}$ are bias vectors. Here, $\left[\begin{array}{c} v_{1}\\ v_{2} \end{array}\right]$ denotes the concatenation of two vectors $v_{1}$ and $v_{2}$, and the softmax function ensures that the probabilities sum up to 1. The initial hidden state $h_{0}$ is set to be a zero vector. We found the best tuned $d_{h}$ to be 128. A dropout rate of 0.1 was applied before the softmax function during training, and the training process was conducted with 100,000 iterations and a learning rate of 0.0005.

Predictions can be made by sampling from the output probabilities of the RNN cells. The sequence generation process stops if an EOS signal is predicted or if the maximum length is reached without EOS signal predicted. Sequences generated in the former case are annotated as “complete”, while those in the latter case as “incomplete”. AMPd‐Up computes a confidence score when generating each sequence. The score is calculated as the geometric mean of probabilities of all predicted symbols in a sequence, including the EOS signal if the sequence is complete. We refer to this score as the “AMPd‐Up score”, and we use it as a measure of confidence of the RNN language model in generating a sequence. In AMPd‐Up, the model is trained multiple times with different random initializations, yielding multiple model instances.

Given one of the 20 possible starting amino acids, the symbol with the highest probability estimated at each time step is taken as the next amino acid prediction (including the EOS signal), resulting in a maximum of 20 candidate AMP sequences generated by a single model instance. In a practical use case, the model will be trained k times and the users would get a candidate AMP list of up to 20k sequences. Assuming we have a non‐convex loss function like most neural network based tasks, different initializations may result in different trained models (Fort et al., 2019), allowing different model instances of AMPd‐Up to capture slightly different aspects of the complex but unknown features of AMPs.

### 4.3 Model evaluation

In order to measure the performance of AMPd‐Up in an efficient and cost‐effective way, we used the predictions from three state‐of‐the‐art *in silico* AMP prediction tools: AMPlify (Li et al., 2022), AMP Scanner Vr.2 (Veltri et al., 2018), and iAMPpred (Meher et al., 2017), as a proxy for AMP sequence generation accuracy. These AMP prediction tools determine whether an input peptide sequence is an AMP or not. Here, the estimated AMP sequence generation accuracy measured by a selected prediction tool was calculated based on the percentage of peptide sequences predicted as AMPs among a generated sequence set. A default setting of balanced model was chosen for AMPlify (v1.1.0) as described in a data note (Li et al., 2023), while the “original production model” was chosen for AMP Scanner Vr.2 on its online server (Veltri et al., 2018). Predictions by iAMPpred were obtained through its online server with its trained model as described in the publication (Meher et al., 2017).

We compared AMPd‐Up with three other AMP sequence generation methods with publicly available models or generated sequences: the LSTM language model (Nagarajan et al., 2018), AMPGAN v2 (Van Oort et al., 2021), and HydrAMP (Szymczak et al., 2022). For each method, a total of 2000 sequences were generated for comparison in five batches. This resulted in five generated sequence sets of 400 sequences for each method. Sequences for the LSTM language model were sampled from the dataset the authors provided (Nagarajan et al., 2018), while those for HydrAMP were obtained through their online server (Szymczak et al., 2022) on November 7, 2022. While all other methods focus on the generation of antibacterial peptides, AMPGAN v2 additionally allows for generating AMP sequences of other function types (e.g., antifungal, antiviral) and the generated sequences are annotated with their predicted functions in the results (Van Oort et al., 2021). For a fairer comparison, only AMPs targeting bacteria were selected for AMPGAN v2. For each AMP sequence generation method measured by each AMP prediction tool, the average estimated accuracy value of the five generated sets was reported, along with the corresponding standard deviation value.

In addition to the estimated sequence generation accuracy, we evaluated the sequences generated by AMPd‐Up based on their amino acid compositions, physicochemical properties, as well as their sequence similarities to the training set and all publicly available known AMPs. The same 2000 sequences generated by AMPd‐Up for performance comparison were used in these analyses.

The properties that cause a peptide sequence to have antimicrobial activity are complex and the mechanisms are still not well understood (Teimouri et al., 2021). Considering the fact that most known AMPs share common characteristics of short lengths and net positive charges (Zhang and Gallo, 2016), we focused on these two important and easy‐to‐calculate physicochemical properties in addition to an amino acid composition analysis.

Moreover, sequence similarities of the AMPd‐Up‐generated sequences to the training set were calculated to evaluate whether the model instances capture high‐level features of AMPs rather than only generating the same or highly similar sequences to the training set. A similar comparison between the AMPd‐Up‐generated sequences and all publicly available known AMPs was done to evaluate the novelty of the generated sequences compared with those known AMP sequences. We note that the training AMPs are antibacterial, while the known AMP sequence set additionally includes those targeting microbes other than bacteria. The known AMP sequence set comprises 4538 distinct sequences that were downloaded from APD3 (Wang et al., 2016) and DADP (Novković et al., 2012) on July 11, 2022 and December 6, 2018, respectively. The similarity between two sequences was calculated as $\left(1-\frac{d_{i,j}}{\max\left(l_{i},l_{j}\right)}\right)\times 100\%$, where $d_{i,j}$ is the edit distance and $l_{i},l_{j}$ are lengths of the sequences regarding the numbers of amino acid residues. The similarity of a sequence to a set of sequences was defined as the maximum of all similarity values calculated between that sequence and the sequences in the target set for comparison (i.e., the similarity of that sequence to its most similar sequence in the target set).

### 4.4 Selecting putative AMPs for validation

To demonstrate the utility of our tool, we trained the model 1000 times, yielding 1000 model instances and 20,000 sequences, 14,188 of which were complete, and 8737 of the complete sequences were distinct. The trained models applied to generate the sequences for validation can be accessed at https://doi.org/10.5281/zenodo.7905591 (Li and Birol, 2023). We define the “count” of a sequence as the number of times it appears in the entire generated set. We further filtered for short sequences with lengths ≤35 aa and obtained 7434 peptide sequences, since shorter peptides are more cost‐effective for synthesis (Lin et al., 2022). We selected 58 of these peptides using different strategies (forming Lists A, B, and C), and validated their bioactivity through *in vitro* experiments (Table 2).

The peptides comprising Lists A and B were chosen following a strategy that stratifies the AMPd‐Up score range of 7434 sequences into same‐length score intervals. For n intervals, each interval can be written as a range from $a+\frac{\left(k-1\right)\left(b-a\right)}{n}$ to $a+\frac{k\left(b-a\right)}{n}$, with k=1,2,…,n and a,b being the minimum and maximum AMPd‐Up scores investigated in the generated sequence set. In our case, a=0.1462 and b=0.3579. All intervals are left‐open and right‐closed, except the first one (k=1) that is closed. If multiple model instances generated the same sequence, the AMPd‐Up score from the first model that generated this sequence was used for stratification. Peptides for List A were sampled by splitting the AMPd‐Up score range of [0.1462, 0.3579] into 40 intervals, and the sequence with top AMPd‐Up score within each interval was chosen. List B peptides were chosen by splitting the same AMPd‐Up score range into five intervals, and then selecting one predicted non‐AMP (as assessed by AMPlify) in each interval with the highest count, or with top AMPd‐Up score if all sequences have the same count in the interval. We note that some intervals did not have any sequences, resulting in 38 sequences in List A and 4 sequences in List B. Additionally, 16 more peptide sequences that appeared with high frequencies (≥40 in sequence counts) in the generated set were selected as List C. All sequences in Lists A and C were predicted as AMPs by AMPlify. In Table 2, we also present the sequence similarity of each sequence to the known AMPs, showing the novelty of those sequences compared with the known AMP sequences.

### 4.5 Antimicrobial susceptibility testing

The antimicrobial activity of our selected peptides was measured in the laboratory by broth microdilution assays to determine the minimum inhibitory and minimum bactericidal concentrations (MICs and MBCs, respectively) as outlined by the Clinical and Laboratory Standards Institute (CLSI) (Clinical and Laboratory Standards Institute, 2015) with some adaptations for testing cationic AMPs as described previously (Wiegand et al., 2008). Laboratory isolates of *E. coli* 25922 and *S. aureus* 29213 were purchased from the American Type Culture Collection (ATCC; Manassas, VA, USA) and were used to test the 58 selected putative AMPs. Bacteria from frozen stocks were streaked onto non‐selective Columbia blood agar with 5% sheep blood (Oxoid) and incubated for 18–24 h at 37°C. The following day, 2–4 colonies were streaked onto a new agar plate and incubated for 18–24 h at 37°C to ensure uniform colony health prior to the assay. A standardized bacterial inoculum was prepared by suspending isolated colonies in Mueller‐Hinton Broth (MHB; Sigma‐Aldrich, St. Louis, MO, USA). The suspension was adjusted to an optical density of 0.08–0.1 at 600 nm, equivalent to a 0.5 McFarland standard of approximately 1–2 × 10^8^ CFU/mL (CFU: colony forming units). The inoculum was then diluted 1:250 to achieve a final concentration of 5 ± 3 × 10^5^ CFU/mL. The target bacterial density was confirmed by examining the total viability counts from the final inoculum.

Candidate AMPs were purchased from and synthesized by GenScript (Piscataway, NJ, USA). These were received in lyophilized format and stored at −20°C, and were suspended in sterile ultrapure water prior to testing. A two‐fold serial dilution of 1280 down to 2.5 μg/mL was prepared in sterile 96‐well polypropylene microtiter plates (Greiner Bio‐One #650261, Kremsmünster, Austria) before the addition of 100 μL of the standardized bacterial inoculum, providing a final AMP testing range of 128 down to 0.25 μg/mL. The MIC values were reported as the lowest peptide concentration where no visible bacterial growth was observed following a 20–24 h incubation at 37°C. For determination of MBC, well contents of the MIC and the two adjacent wells containing the two‐ and four‐fold higher peptide concentrations were plated onto non‐selective nutrient agar. The concentration in which 99.9% of the inoculum were killed after an incubation for 24 h at 37°C was reported as the MBC.

A known AMP Ranatuerin‐4 (Goraya et al., 1998) from the American bullfrog and an in‐house peptide [TKPKG]_3_ (OT15) were used as the positive and negative control peptides, respectively. We note that OT15 was truncated and derived from a negative control peptide [TKPKG]_4_ (OT20), which, while not antimicrobial, shares similar characteristics with AMPs and has been used in previous studies (Horváti et al., 2017).

### 4.6 Hemolysis assay

The toxicity of the selected peptides to RBCs was evaluated by hemolysis experiments. Whole blood from healthy donor pigs was purchased from Lampire Biological Laboratories (Pipersville, PA, USA). RBCs were washed and isolated by centrifugation using Roswell Park Memorial Institute (RPMI) medium (Life Technologies, Grand Island, NY, USA). All centrifugation steps were performed at 500× *g* for 5 min in an Allegra‐6R centrifuge (Beckman Coulter, CA, USA). Peptides were suspended and serially diluted from 1280 down to 10 μg/mL using RPMI medium in a 96‐well polypropylene microtiter plate, and then they were combined with 100 μL of the 1% RBC solution. This resulted in a final AMP testing range of 128 down to 1 μg/mL. Following an incubation at 37°C for 30–45 min, plates were centrifuged and a 1/2 volume from each supernatant was transferred to a new 96‐well plate. The absorbance of the wells was measured at 415 nm utilizing the Cytation 5 Cell Imaging Multimode Reader (BioTek, CA, USA); the peptide concentration that lysed 50% of the RBCs (HC_50_) was used to report the hemolytic activity. Absorbance readings from wells containing RBCs treated with 11 μL of a 2% Triton‐X100 solution or RPMI medium (AMP solvent‐only) were used to define 100% and 0% hemolysis, respectively.

## AUTHOR CONTRIBUTIONS

**Chenkai Li:** Conceptualization; writing – original draft; methodology; software; investigation; data curation; visualization; formal analysis; writing – review and editing; validation. **Darcy Sutherland:** Formal analysis; validation; investigation; writing – review and editing; methodology. **Amelia Richter:** Formal analysis; validation; investigation; writing – review and editing; methodology. **Lauren Coombe:** Formal analysis; writing – review and editing. **Anat Yanai:** Formal analysis; validation; investigation; writing – review and editing; methodology. **René L. Warren:** Formal analysis; investigation; writing – review and editing. **Monica Kotkoff:** Project administration; writing – review and editing. **Fraser Hof:** Conceptualization; funding acquisition; writing – review and editing; supervision. **Linda M. N. Hoang:** Conceptualization; funding acquisition; writing – review and editing; supervision. **Caren C. Helbing:** Conceptualization; funding acquisition; writing – review and editing; supervision. **Inanc Birol:** Conceptualization; funding acquisition; supervision; methodology; software; formal analysis; investigation; writing – review and editing.

## CONFLICT OF INTEREST STATEMENT

IB is a co‐founder of and executive at Amphoraxe Life Sciences Inc.

## Supporting information


**Data S1.** Supporting information.
