# Supplementary material for: De novo synthetic antimicrobial peptide design with a recurrent neural network
Source: Protein Sci. 2024 Jul 11;33(8):e5088. doi: 10.1002/pro.5088 (PMC11237553; doi:10.1002/pro.5088)
Supplement: Supplementary file 1 — Data S1. Supporting information. [file PRO-33-e5088-s001.pdf]

Supporting Information

De novo synthetic antimicrobial peptide design with a recurrent neural network

Chenkai Li[1, 2], Darcy Sutherland[1, 3, 4], Amelia Richter[1, 3], Lauren Coombe[1], Anat Yanai[1, 3], René L. Warren[1], Monica Kotkoff[1], Fraser Hof[5], Linda M.N. Hoang[3, 4], Caren C. Helbing[6], and Inanc Birol[1, 3, 4, 7, *]

[1] Canada's Michael Smith Genome Sciences Centre, BC Cancer Agency, Vancouver, BC, V5Z 4S6, Canada
[2] Bioinformatics Graduate Program, University of British Columbia, Vancouver, BC, V6T 1Z4, Canada
[3] Public Health Laboratory, British Columbia Centre for Disease Control, Vancouver, BC, V5Z 4R4, Canada
[4] Department of Pathology and Laboratory Medicine, University of British Columbia, Vancouver, BC, V6T 1Z4, Canada
[5] Department of Chemistry and the Centre for Advanced Materials and Related Technology, University of Victoria, Victoria, BC, V8W 3V6, Canada
[6] Department of Biochemistry and Microbiology, University of Victoria, Victoria, BC, V8P 5C2, Canada
[7] Department of Medical Genetics, University of British Columbia, Vancouver, BC, V6H 3N1, Canada

* Correspondence: Inanc Birol (ibirol@bcgsc.ca)

Supplementary Figures

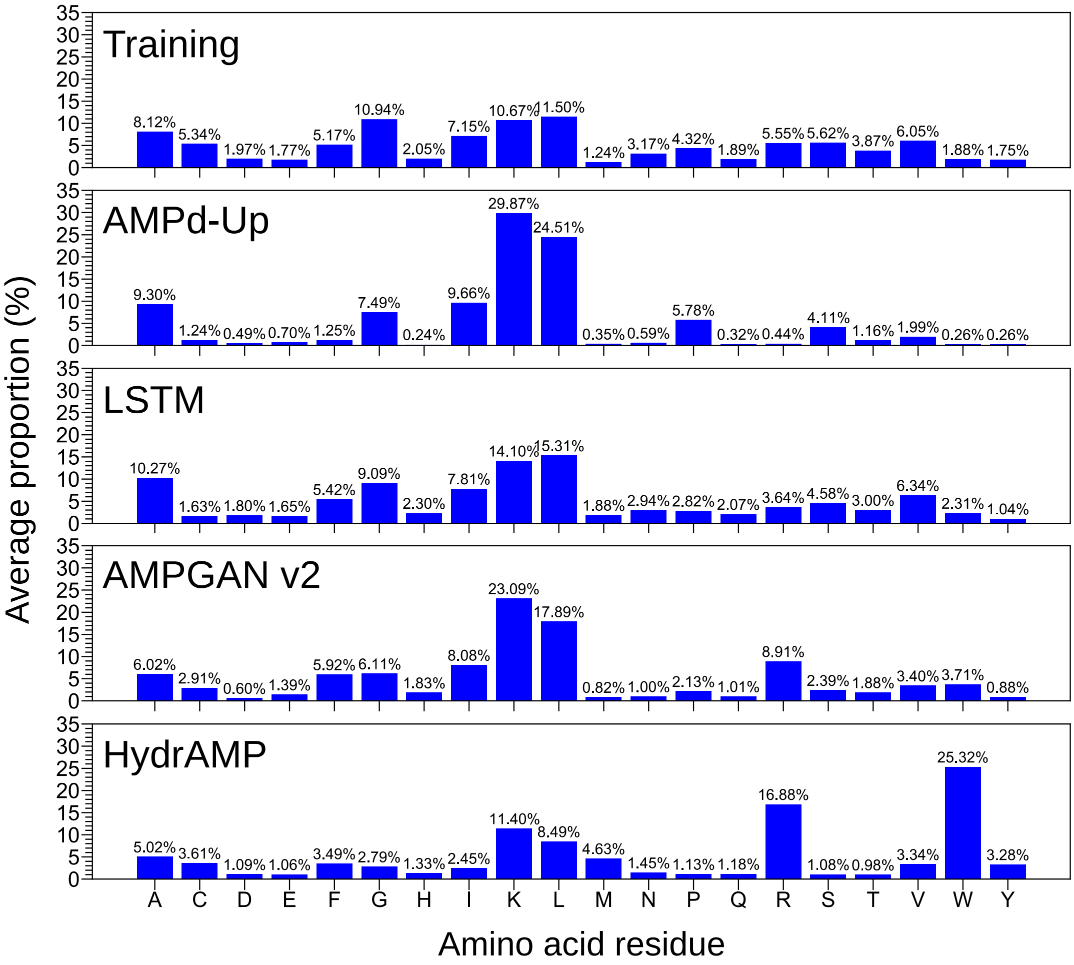

Figure S1: Amino acid compositions of sequences generated by different AMP sequence generation methods. For each AMP sequence generation method (Nagarajan et al. 2018; Van Oort et al. 2021; Szymczak et al. 2022), the average proportions of different amino acid residues per peptide sequence were calculated. The amino acid compositions of the AMPd-Up training sequences were also analyzed for comparison.

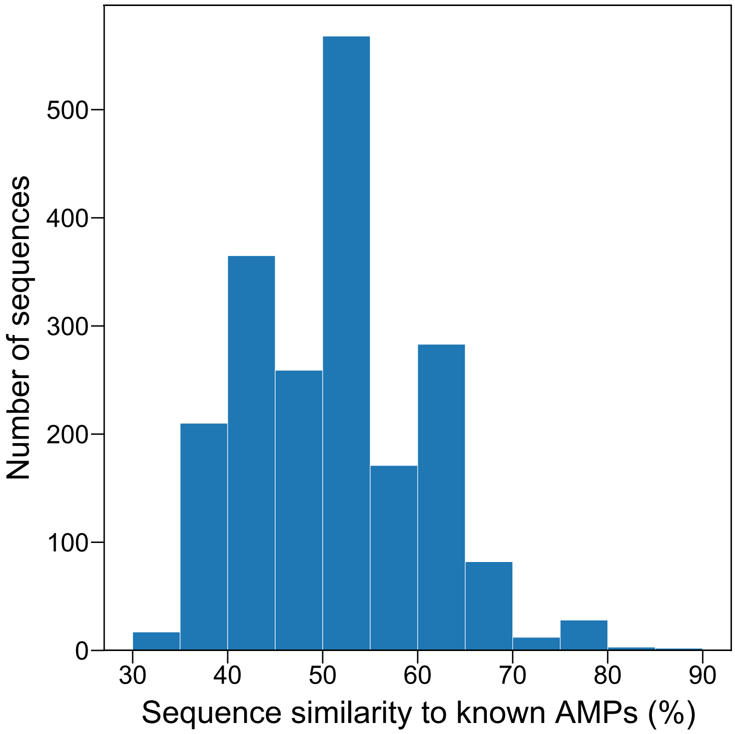

Figure S2: Sequence similarity distribution of the AMPd-Up-generated sequences to known AMPs. The known AMP sequence set comprises 4,538 distinct sequences downloaded from Antimicrobial Peptide Database (APD3) (Wang et al. 2016) and Database of Anuran Defense Peptides (DADP) (Novković et al. 2012). The sequence similarity distribution, with a mean of 51.03% and a standard deviation of 9.38%, was calculated based on the 2,000 sequences generated by AMPd-Up. The sequence similarity of each generated sequence to known AMPs was considered as the similarity of that sequence to its most similar known AMP sequence, based on which the distribution was plotted.

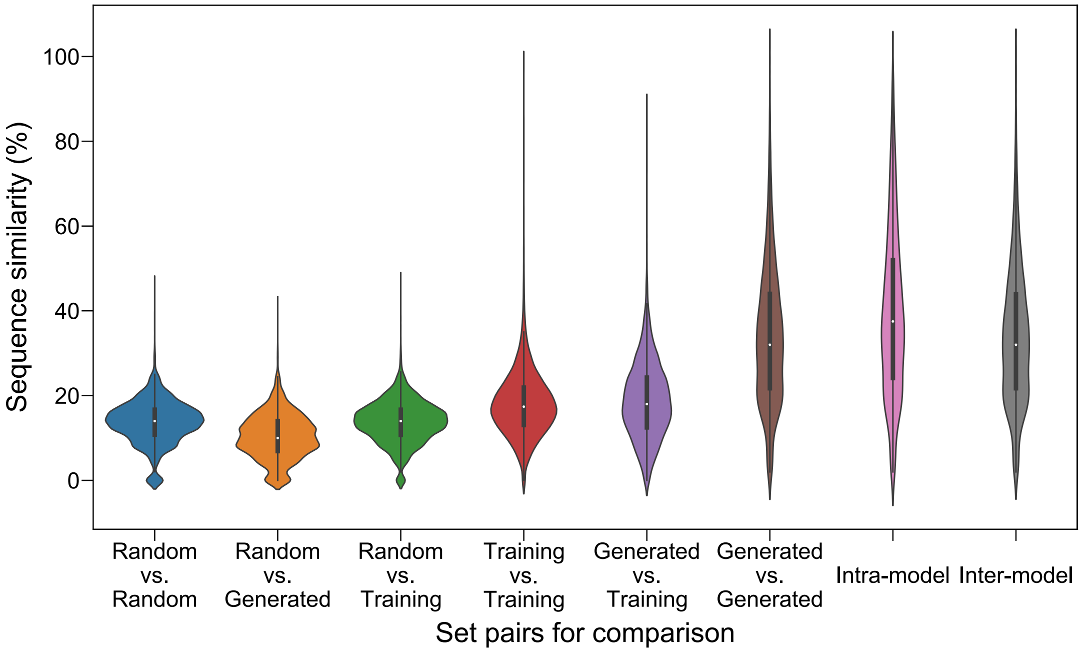

Figure S3: Distributions of pairwise sequence similarities between different sequence sets of AMPd-Up. The pairwise sequence similarities between two different sets of sequences were calculated as the similarities of all sequence pairs between the two sets (e.g., Generated vs. Training), while the pairwise sequence similarities of the same set of sequences (i.e., sequence diversity measurements) were defined as the similarities of sequences to each other in the set (e.g., Generated vs. Generated). The intra-model sequence similarities were calculated as the similarities of sequences generated by the same model instance to each other, while inter-model sequence similarities were calculated as pairwise sequence similarities between sets of sequences generated by different model instances. A set of 2,000 random sequences matching the length distribution of the 2,000 generated sequences were added for comparison, in addition to the training and generated sequence sets. Mean (μ) and standard deviation (σ) values of each distribution are as follows: Random vs. Random (μ = 13.71%, σ = 5.09%), Random vs. Generated (μ = 10.34%, σ = 5.28%), Random vs. Training (μ = 13.76%, σ = 4.91%), Training vs. Training (μ = 18.06%, σ = 7.92%), Generated vs. Training (μ = 18.80%, σ = 8.94%), Generated vs. Generated (μ = 33.61%, σ = 16.18%), Intra-model (μ = 39.14%, σ = 19.79%), and Inter-model (μ = 33.56%, σ = 16.14%). Two-sided Kolmogorov-Smirnov tests reveal that the difference between any two of the distributions is significant ($p < 0.0018$), except that between Generated vs. Generated and Inter-model ($p = 0.0519$). We note that 0.0018 is an adjusted alpha level calculated with Šidák correction (Šidák 1967) from a family-wise alpha level of 0.05 for the multiple comparisons.

Supplementary Tables

Table S1: Antimicrobial susceptibility testing and hemolysis experiment results of the 58 selected peptides *in vitro*. Peptides were tested for their antimicrobial activity against *Escherichia coli* ATCC 25922 and *Staphylococcus aureus* ATCC 29213 for their minimum inhibitory concentration (MIC) and minimum bactericidal concentration (MBC) values. Porcine red blood cells (RBCs) were used to test the hemolytic activity of the selected peptides for their hemolytic concentration (HC_{50}) values. Data are presented as the lowest effective peptide concentration range (µg/mL) observed in three independent experiments performed in duplicate, with one maximum data point and one minimum data point dropped for each measurement. Ranateurin-4 (Goraya et al. 1998) and OT15 were used as the positive and negative control peptides, respectively.

List	Peptide name	Antimicrobial susceptibility testing				Hemolysis testing
		E. coli ATCC 25922		*S. aureus* ATCC 29213		Porcine RBCs
		MIC (µg/mL)	MBC (µg/mL)	MIC (µg/mL)	MBC (µg/mL)	HC_{50} (µg/mL)
A	DeNo1001	2 – 4	2 – 4	32 – 128	64 – >128	>128
	DeNo1002	2 – 4	2 – 4	64 – 128	64 – >128	128
	DeNo1003	2 – 4	2 – 4	≥128	≥128	>128
	DeNo1004	64	64	>128	>128	>128
	DeNo1005	>128	>128	>128	>128	>128
	DeNo1006	8 – 16	16 – >32	>128	>128	>128
	DeNo1007	4	4 – 8	4	4 – 8	>128
	DeNo1008	>128	>128	>128	>128	16 – 32
	DeNo1009	8 – 16	8 – >128	>128	>128	>128
	DeNo1010	4	4 – 8	32 – 64	32 – 128	64 – 128
	DeNo1011	32	32	>128	>128	32
	DeNo1012	32	64	>128	>128	>128

	DeNo1013	16	16 – 32	>128	>128	64
	DeNo1014	64	64	>128	>128	≥128
	DeNo1015	128	128	>128	>128	>128
	DeNo1016	4	4	2 – 4	2 – 8	4 – 8
	DeNo1017	4	4	2 – 4	2 – 4	16
	DeNo1018	1 – 2	2 – 4	8	8	128
	DeNo1019	16	16	>128	>128	>128
	DeNo1020	64 – 128	64 – 128	>128	>128	>128
	DeNo1021	8	8 – 16	32 – 64	32 – 128	>128
	DeNo1022	4	16 – >128	4	4 – 16	≥128
	DeNo1023	128	128	>128	>128	>128
	DeNo1024	≥128	≥128	>128	>128	>128
	DeNo1025	>128	>128	>128	>128	>128
	DeNo1026	16 – 32	32 – 64	≥128	≥128	≥128
	DeNo1027	32	32 – 64	>128	>128	>128
	DeNo1028	>128	>128	>128	>128	>128
	DeNo1029	>128	>128	>128	>128	>128
	DeNo1030	128	≥128	>128	>128	>128
	DeNo1031	8 – 16	16	16	32 – >64	128
	DeNo1032	>128	>128	>128	>128	>128
	DeNo1033	>128	>128	>128	>128	>128
	DeNo1034	16 – 32	16 – 64	>128	>128	>128
	DeNo1035	>128	>128	>128	>128	>128
	DeNo1036	>128	>128	>128	>128	>128
	DeNo1037	>128	>128	>128	>128	>128

	DeNo1038	64	64 – 128	>128	>128	>128
B	DeNo1039	>128	>128	>128	>128	32 – 64
	DeNo1040	64 – 128	≥128	64	64 – 128	>128
	DeNo1041	>128	>128	>128	>128	>128
	DeNo1042	>128	>128	>128	>128	>128
C	DeNo1043	>128	>128	>128	>128	>128
	DeNo1044	≥128	≥128	>128	>128	>128
	DeNo1045	64	64 – 128	>128	>128	>128
	DeNo1046	16 – 64	16 – 64	128	128	>128
	DeNo1047	≥128	≥128	>128	>128	>128
	DeNo1048	128	128	>128	>128	>128
	DeNo1049	4 – 8	4 – 16	>128	>128	>128
	DeNo1050	>128	>128	>128	>128	>128
	DeNo1051	16 – 32	16 – 64	>128	>128	>128
	DeNo1052	≥128	≥128	>128	>128	>128
	DeNo1053	>128	>128	>128	>128	>128
	DeNo1054	>128	>128	>128	>128	>128
	DeNo1055	≥128	≥128	>128	>128	>128
	DeNo1056	32 – >128	32 – >128	>128	>128	>128
	DeNo1057	8 – 16	8 – 32	32	32 – 128	>128
	DeNo1058	>128	>128	>128	>128	>128
Controls	Ranateurin-4	8 – 16	8 – 16	2 – 4	4	64 – 128
	OT15[a]	>128	>128	>128	>128	>128

[a] OT15 (TKPKGTKPKGTKPKG) is a truncated form of a negative control peptide OT20 (Horváti et al. 2017) used in previous studies.

References

Goraya J, Knoop FC, Conlon JM. 1998. Ranatuerins: Antimicrobial Peptides Isolated from the Skin of the American Bullfrog,Rana catesbeiana. Biochem Biophys Res Commun. 250(3):589–592. doi:10.1006/bbrc.1998.9362.

Horváti K, Bacsa B, Mlinkó T, Szabó N, Hudecz F, Zsila F, Bősze S. 2017. Comparative analysis of internalisation, haemolytic, cytotoxic and antibacterial effect of membrane-active cationic peptides: aspects of experimental setup. Amino Acids. 49(6):1053–1067. doi:10.1007/s00726-017-2402-9.

Nagarajan D, Nagarajan T, Roy N, Kulkarni O, Ravichandran S, Mishra M, Chakravortty D, Chandra N. 2018. Computational antimicrobial peptide design and evaluation against multidrug-resistant clinical isolates of bacteria. J Biol Chem. 293(10):3492–3509. doi:10.1074/jbc.M117.805499.

Novković M, Simunić J, Bojović V, Tossi A, Juretić D. 2012. DADP: the database of anuran defense peptides. Bioinformatics. 28(10):1406–1407. doi:10.1093/bioinformatics/bts141.

Van Oort CM, Ferrell JB, Remington JM, Wshah S, Li J. 2021. AMPGAN v2: Machine Learning-Guided Design of Antimicrobial Peptides. J Chem Inf Model. 61(5):2198–2207. doi:10.1021/acs.jcim.0c01441.

Šidák Z. 1967. Rectangular Confidence Regions for the Means of Multivariate Normal Distributions. J Am Stat Assoc. 62(318):626–633. doi:10.1080/01621459.1967.10482935.

Szymczak P, Możejko M, Grzegorzek T, Bauer M, Neubauer D, Michalski M, Sroka J, Setny P, Kamysz W, Szczurek E. 2022. HydrAMP: a deep generative model for antimicrobial peptide discovery. bioRxiv. doi:10.1101/2022.01.27.478054. https://www.biorxiv.org/content/10.1101/2022.01.27.478054v1.

Wang G, Li X, Wang Z. 2016. APD3: the antimicrobial peptide database as a tool for research and education. Nucleic Acids Res. 44(D1):D1087–D1093. doi:10.1093/nar/gkv1278.
